# Human primary motor cortex is both activated and stabilized during observation of other person's phasic motor actions

**DOI:** 10.1098/rstb.2013.0171

**Published:** 2014-06-05

**Authors:** Riitta Hari, Mathieu Bourguignon, Harri Piitulainen, Eero Smeds, Xavier De Tiège, Veikko Jousmäki

**Affiliations:** 1 Brain Research Unit, O.V. Lounasmaa Laboratory, Aalto University, 00076 AALTO, Espoo, Finland; 2 MEG Core and AMI Centre, Aalto NeuroImaging, Aalto University School of Science, Aalto University, 00076 AALTO, Espoo, Finland; 3 Laboratoire de Cartographie fonctionnelle du Cerveau, UNI—ULB Neuroscience Institute, Université libre de Bruxelles (ULB), 1070 Brussels, Belgium

**Keywords:** motor cortex, brain rhythms, inhibition, imitation

## Abstract

When your favourite athlete flops over the high-jump bar, you may twist your body in front of the TV screen. Such automatic motor facilitation, ‘mirroring’ or even overt imitation is not always appropriate. Here, we show, by monitoring motor-cortex brain rhythms with magnetoencephalography (MEG) in healthy adults, that viewing intermittent hand actions of another person, in addition to activation, phasically stabilizes the viewer's primary motor cortex, with the maximum of half a second after the onset of the seen movement. Such a stabilization was evident as enhanced cortex–muscle coherence at 16–20 Hz, despite signs of almost simultaneous suppression of rolandic rhythms of approximately 7 and 15 Hz as a sign of activation of the sensorimotor cortex. These findings suggest that inhibition suppresses motor output during viewing another person's actions, thereby withholding unintentional imitation.

## 1. Introduction

Viewing another person may trigger an unconscious urge to imitate their actions or postures. Some motor actions are highly contagious, but excessive imitation is socially inappropriate and disadvantageous for normal adult behaviour.

We have previously shown that seeing another person's motor acts activates the viewer's primary motor (M1) cortex, evident from the suppression of the approximately 20 Hz motor-cortex rhythm [1]; the motor-cortex activation during observation is, however, considerably weaker than that during own actions. Executed and observed movements are commonly considered to be associated with activations in overlapping motor brain areas that form nodes of ‘mirroring systems’ (for reviews, see e.g. [2,3]), and one may thus wonder how it is possible to prevent imitation of every seen action.

In fact, some neurological patients, mainly suffering from frontal-lobe lesions, may become ‘echopractic’, imitating basically every action they see without being instructed so. The mechanisms of inhibition of undesired imitation are still largely unknown, although they, on the basis of patient studies, likely reflect released inhibition by the frontal lobes to more posterior, mainly parietal brain areas [4].

Bien *et al*. [5] in a combined functional magnetic resonance imaging (fMRI) and transcranial magnetic stimulation (TMS) study in healthy subjects provided brain-level support for the idea [4] that automatic and intentional imitation have different neuronal bases. In their model, Bien and co-workers proposed that the right premotor cortex is involved in automatic imitation and the input it receives from the right middle/inferior frontal lobe leads to inhibition of imitation. The other regions in the circuitry were assumed to be the parietal cortices in both hemispheres and the left opercular area [5]. However, this study still left open the effects of inhibition on the final effector, the corticospinal pathway.

During simple isometric contraction, corticospinal communication can be monitored by cortex–muscle coherence (CMC) occurring at approximately 20 Hz during weak contraction [6,7]. Although intracranial recordings imply generation of CMC in several cortical areas, the CMC picked up with magnetoencephalography (MEG) seems to mainly reflect activity in the M1 cortex, minimally affected by peripheral afferents (see Discussion). The CMC recorded during isometric contraction thus seems to be a suitable non-invasive tool to monitor the maintenance of the descending corticospinal drive from M1 cortex to the spinal motoneuron pool [6–11].

In this study, we used the ∼20 Hz CMC, with MEG recordings, to find signs in the human M1 cortex of inhibition of inappropriate imitation. It is already known that during own actions, such as ramp movements, the ∼20 Hz CMC is abolished or strongly suppressed [8].

In practice, subjects were asked to keep a steady contraction between the forefinger and thumb while they were viewing intermittent brief pinching movements of the experimenter. Variations in the strength of the CMC were expected to reflect changes in the functional state and stability of the M1 cortex, informing whether seeing another person's movements would automatically modify the descending cortex–motoneuron drive. By comparing CMC modulation with the changes in mu-rhythm power, we found evidence for a dual effect in the primary motor cortex: one neuronal population involved in the stabilization and another in the activation of the M1 cortex in overlapping time windows within the 1 s interval following the onset of the observed movement.

## 2. Material and methods

### (a) Subjects

Fourteen healthy subjects (8 female and 6 male; age 20–38 years, mean 28.6 years) were studied. On the Edinburgh Handedness Inventory scale [9] from −100 (left) to +100 (right), 13 subjects were right-handed (range 67–100) and one ambidextrous (−20). The study had prior approval by the ethics committee of the Helsinki and Uusimaa hospital district, and the subjects gave written informed consent before participation. Subjects were compensated monetarily for lost working hours and travel expenses.

### (b) Experimental protocol

**Figure 1.**
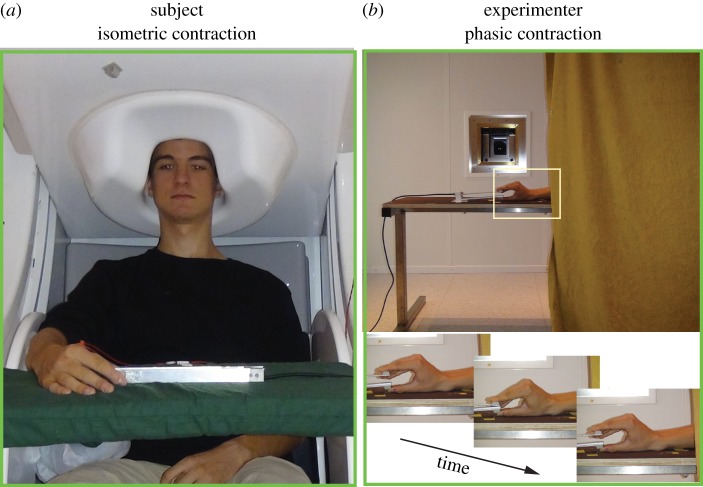
Experimental set-up. Subject (*a*) maintained steady isomeric contraction against a force transducer while observing intermittent phasic pinching movements performed by an experimenter (*b*) whose hand was visible in front of the subject. The right panels show the subject's point of view.

Figure 1 shows the experimental set-up with maintained isometric contraction, performed by the subject (left panel), and intermittent phasic contractions performed simultaneously by the experimenter (right panels). Subjects were sitting in an upright position with the left hand on the thigh and the right hand lying on a table in front of them, the scalp being covered by the helmet of the MEG device. Earplugs were used to reduce concomitant auditory noise while the subjects maintained isometric contraction against a force transducer positioned between the right thumb and index finger; data were collected from each subject during two sessions, 5 min each. Prior to MEG recordings, the maximum isometric voluntary contraction between right thumb and index finger was measured for each subject. During the MEG recordings, the isometric force exerted by the subject was kept within 10 ± 2% of maximum by presenting auditory feedback (1 kHz tone) whenever the force level stepped out of this range.

An experimenter sitting behind a curtain held an identical force transducer and kept it between his right thumb and index finger in the same way as instructed to the subjects. The experimenter's hand and the force transducer were on a table (67 cm in height) 2 m in front of the subject. Only the experimenter's hand and a part of his forearm were visible to the subjects (figure 1, right panels). The experimenter performed self-paced intermittent (once every 3–6 s) dynamic contractions, pinching with index finger and thumb the aluminium handles of the force transducer. The experimenter's contractions lasted for about 500 ms and were strong enough to reach the maximum motion range (1.9 cm) of the compliant force transducer. The force signal was used to evaluate the displacement between the fingers. Subjects were instructed to keep their gaze on the experimenter's hand.

### (c) Measurements

Brain activity was recorded in a magnetically shielded room (Imedco AG, Hägendorf, Switzerland) with a 306 channel whole-scalp neuromagnetometer (Elekta Neuromag, Elekta Oy, Helsinki, Finland). The recording passband was 0.1–330 Hz and the signals were sampled at 1 kHz. The measurements were carried out at the MEG Core of Aalto NeuroImaging at Aalto University. The position of the subject's head inside the MEG helmet was continuously monitored by feeding current to four head-tracking coils located on the scalp. The locations of the coils and 156–292 head-surface (scalp, nose) points were digitized with respect to anatomical fiducials with an electromagnetic tracker (Fastrak, Polhemus, Colchester, VT, USA). MEG and segmented MRI coordinate systems were coregistered using the three anatomical fiducial points for the initial estimation and the head-surface points to manually refine the surface coregistration.

Surface electromyography (EMG) was recorded between two electrodes (separated by approx. 1 cm) over the right first dorsal interosseous muscle. The recording passband was 0–330 Hz for force transducer output and 0.1–330 Hz for EMG, and both signals were digitized at 1 kHz.

Three-dimensional T1 MRIs of the brain were acquired with whole-body General Electric Signa VR 3.0 T MRI scanner (Signa VH/i, General Electric, Milwaukee, WI, USA) at the AMI Centre of Aalto NeuroImaging, Aalto University.

### (d) Data analysis and statistical evaluation

Continuous MEG data were first preprocessed offline using the signal-space separation method to suppress external interferences and to correct for head movements [10].

Static and dynamic phenomena were analysed separately, as will be presented below.

#### (i) Static phenomena

Continuous data from both sessions were split into 1024 ms epochs with 819 ms epoch overlap (see [11]), leading to a frequency resolution of approximately 1 Hz. Epochs with MEG signals (filtered from 1 to 195 Hz, with additional notch filters at 50 Hz and its harmonics) exceeding 3 pT in magnetometer channels or 0.7 pT cm^−1^ in gradiometer channels were rejected to avoid contamination by eye movements, muscle activity and other artefacts. Filtered EMG signals (passband 20–195 Hz with notch filters at 50 Hz and harmonics) were rectified, and used in coherence computation with all MEG signals.

Previous MEG research shows that during low-force isometric contraction, CMC usually peaks at 10–30 Hz [6,7]. For further analysis, we thus selected—from a predefined subset of 18 gradiometers covering the left rolandic area of each subject—the gradiometer with the strongest coherence in this frequency range. Subjects lacking statistically significant CMC (see below) were removed from further analysis.

The statistical significance of coherence level was based on surrogate data [12]. This approach overcomes the multiple-comparison issue, which has no straightforward analytical solution for highly dependent time series, such as MEG signals. First, 1000 surrogate coherence spectra were computed between real MEG signals and Fourier transform surrogate EMG signals. The Fourier transform surrogate imposes the power spectrum to remain the same as in the original signal but replaces the phase of Fourier coefficients by random numbers in the range [−*π*; *π*] in the surrogate signals [12]. Then, the maximum value across the preselected 18 gradiometers and the 10–30 Hz frequency range was extracted for each surrogate coherence spectrum to compute the cumulative density function of the maximum coherence occurring owing to stochastic matching between EMG and MEG signals. Coherence thresholds at *p* < 0.05, corrected for multiple comparisons, were then considered as the 95-percentiles of the corresponding cumulative density functions.

#### (ii) Dynamic phenomena

Artefact-free trials were extracted from −2511 to 3512 ms relative to experimenter's movement onset, defined as the moment when the displacement of the force transducer exceeded 0.9 cm. We rejected all trials in which the subject's contraction had not been steady, defined as epochs in which the ratio between the mean and standard deviation of the force signal (low-pass filtered at 5 Hz) was less than 25. Power and coherence spectra were then computed in the 0–45 Hz range between the selected MEG signal and rectified EMG in every 1024 ms window sliding in 100 ms steps from −2000 to 3000 ms. For each subject, this procedure yielded a time–frequency MEG power, cross-spectral and coherence maps with 51 time steps and 47 frequency bins. Relative MEG power maps were further computed by normalizing the MEG power maps by the mean baseline MEG power from −2000 to −500 ms. Group-level coherence and relative MEG power maps were then produced by averaging these maps across subjects.

Statistical evaluation of time–frequency maps was again based on surrogate data, using the original MEG and EMG signals but replacing the experimenter's movement onsets with a series of dummy onsets. The first dummy onset was randomly chosen in a 3 s interval centred on the experimenter's first movement onset; the remaining dummy-onsets series was constructed from the randomly shuffled times between all consecutive movement onsets. These surrogate data had the same properties (power spectra and coherence spectra) as the original data except that they were no longer linked to the experimenter's movement onsets. For all subjects, 1000 surrogate power, cross-spectral and coherence time–frequency maps were computed with 1000 different series of dummy onsets.

These surrogate data were used to assess the existence of statistically significant clusters of increased or decreased coherence. First, a threshold for statistically significant group-level coherence increase (or decrease) was computed for each resel (resolution element is the equivalent of pixels in time–frequency maps, and a cluster is a set of adjacent resels) as the 95-percentile (or 5-percentile) of the surrogate coherence value in this resel. Clusters of group-level coherence above (or below) this resel-specific threshold were then extracted in the −500 to 2000 ms window. Finally, to assess the statistical significance of these clusters, the same clustering analysis was performed with the group-level surrogate coherence maps to extract the 97.5-percentile of the maximal cluster size. A cluster with a size above this 97.5-percentile corresponds to a statistically significant increase (or decrease) of coherence at *p* < 0.05 (Bonferroni corrected for the two comparisons).

The temporal changes of the relative MEG power as well as the existence of clusters of increased or decreased relative MEG power were assessed similarly.

Stability of the subjects' isometric force and muscle activity during the analysis period was assessed using the same surrogate dataset. For each subject, force and EMG power signals were averaged across trials, low-pass filtered at 5 Hz and normalized by their baseline value assessed in the –2000 to −500 ms range. Maximum variation in the −500 to 2000 ms range was compared with values obtained with the above-described surrogate data for group-level force and EMG signals.

#### (iii) Source reconstruction

Individual MRIs were segmented using Freesurfer software (Martinos Center for Biomedical Imaging, MA, USA). Then, the MEG forward model was computed for two orthogonal tangential current dipoles placed on a homogeneous 5 mm grid source space that covered the whole brain (MNE suite; Martinos Center for Biomedical Imaging). Individual coherence maps as well as individual normalized power maps (baseline from −2000 to −500 ms) were then produced within the computed source space using the dynamic imaging of coherent sources approach with minimum-variance beamformer [13–15]. Following this, nonlinear transformation from individual MRIs to the standard Montreal Neurological Institute brain was computed using the spatial-normalization algorithm implemented in Statistical Parametric Mapping (SPM8; Wellcome Department of Cognitive Neurology, London, UK) and applied to individual maps. Finally, the group-level maps were obtained by averaging normalized maps across subjects.

## 3. Results

**Figure 2.**
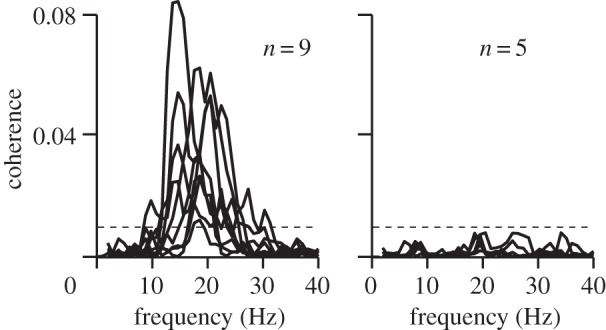
Coherence spectra from the MEG sensor that displayed the highest CMC in each individual. The horizontal dashed line corresponds to the coherence threshold at *p* = 0.05 corrected for multiple comparisons. Spectra are superimposed separately for the nine subjects showing statistically significant coherence (left) and for the five subjects with no statistically significant coherence (right).

All subjects were able to maintain the isometric contraction for the two 5-min recording sessions. Figure 2 shows coherence spectra for all 14 subjects, presented in a way that the statistically significant (*p* < 0.05) spectra of nine subjects (four female and five male) and the statistically non-significant spectra of five subjects are superimposed separately. The significance level was corrected for multiple comparisons (18 sensors and 20 frequency bins). Further analysis was based on the statistically significant data of the nine subjects with statistically significant CMC.

**Figure 3.**
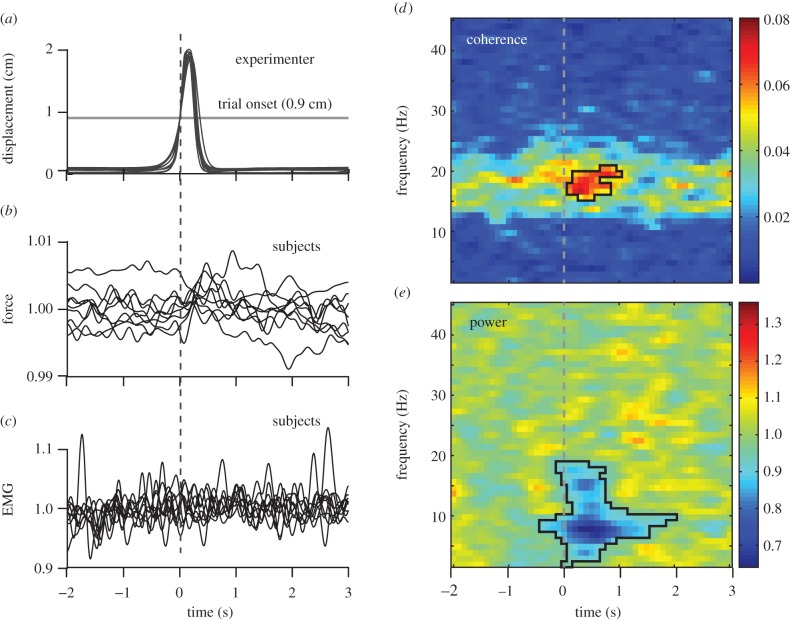
(*a*) Experimenter's displacement curves averaged over trials of each subject's experiment. The vertical line indicates the movement onset defined as displacement exceeding 0.9 cm. (*b*,*c*) Over-trial averaged isometric force and EMG normalized by their mean for each subject. One line is displayed per subject. (*d*) Group-level (*n* = 9) time–frequency map for coherence, colour-coded as a function of frequency from −2 to 3 s with respect to the onset of experimenter's movement. The statistically significant (*p* < 0.05) time–frequency cluster of increased and decreased coherence value is outlined with a thin black line. (*e*) Similar visualization for the relative MEG power.

Figure 3 summarizes the peripheral measures, group-level coherence and MEG power as a function of time. Figure 3*a* shows the average displacement of the experimenter's pinching movements superimposed for the experiment of each subject. The close similarity of the traces indicates that the experimenter's movements, serving as visual stimuli for the subjects, remained consistent across subjects. Figure 3*b* shows the force and figure 3*c* the surface EMG from the first dorsal interosseous muscle for each individual. These recordings indicate that the subjects were able to maintain the steady isometric contraction with no statistically significant modulation in relation to the experimenter's movements (*p* = 0.28 for force and *p* = 0.19 for EMG). Note that the subjects were able to perceive the brief preparation of the movement before time zero as the movement onset was defined as the time when the displacement exceeded 0.9 cm (maximum 1.9 cm).

Figure 3*d* shows the group-level time–frequency map for coherence and figure 3*e* the map for relative MEG power. A statistically significant (*p* < 0.001) cluster of coherence increase comprised 36 resels centred on [500 ms; 18 Hz], with time ranging from 100 to 1000 ms, and frequency from 16 to 21 Hz. In other words, seeing the movements performed by the experimenter increased the subject's CMC level within the first second.

The relative MEG power (figure 3*e*) was reduced below 20 Hz immediately after movement onset, with the strongest effects around 7 Hz and, to a smaller extent, around 15 Hz. The statistically significant cluster of power decrease comprised 181 resels centred on [644 ms; 10.0 Hz] (time range from −300 to 2100 ms, frequency range 2.0–18.6 Hz; *p* < 0.001).

**Figure 4.**
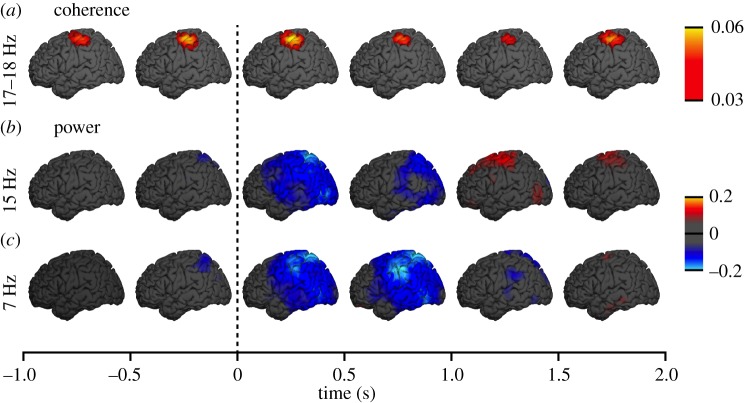
Source reconstruction at the group level (*n* = 9) separately for the coherence (*a*) and relative MEG power (*b*,*c*). Coherence was computed at 17–18 Hz, and power was computed at 7 and 15 Hz.

Figure 4 shows the group-level source reconstructions for coherence and MEG power on the left hemisphere from 1 s before to 2 s after the movement onset. The coherence is increased (yellowing blobs in figure 4*a*) after the movement in the sensorimotor cortex, whereas the power suppression (blue in figure 4*c*) has a different and wider distribution, involving both sensorimotor cortices and parieto-occipital regions.

## 4. Discussion

### (a) Main results

We have shown, by monitoring the level of cortical MEG rhythms and CMC, that the human sensorimotor cortex reacts to another person's intermittent brief pinching movements in two apparently opposite ways within overlapping time windows: first, in agreement with previous results, the level of rhythmic activity *decreases*, at group level at frequencies from 7 to 18 Hz, within the first second of the experimenter's movement. Second, as a novel finding, the CMC *increases* phasically at 16–20 Hz, with a peak about 500 ms after the experimenter's movement onset. It is important to note that the decrease in rhythmic activity is of the same direction as what occurs during the subject's own movements (e.g. [1]), whereas the increase in CMC is opposite to what is observed during the subject's own actions, such as ramp movements, when the CMC either disappears or is strongly suppressed (e.g. [8]). To understand the functional role of these effects, we discuss first some observations on human M1-cortex oscillations and then consider the characteristics of CMC.

#### (i) Motor-cortex oscillations

At rest, the rolandic mu rhythm is characterized by two main frequencies that have nearly, but not exactly, harmonic relationship and differ in the details of time courses [16,17]. These two frequency components appear to be related to separate functional networks: the ∼10 Hz rhythm reflects predominantly (but not exclusively) somatosensory cortical function, while the ∼20 Hz rhythm is mainly associated with motor-cortex function [17,18].

The bulk of evidence indicates that the ∼20 Hz component of the mu rhythm—visible in intracranial recordings, in scalp EEG as well as in MEG—is suppressed during movements [17,19–22], during preparation of movement [23], and even during motor imagery [24] and action observation [1]. In other words, suppression of motor-cortex rhythmic activity is likely related to excitation of the cortex.

This inverse relationship between oscillatory activity and excitability of the M1 cortex is also supported by TMS findings [25] demonstrating decreased motor-cortex excitability during the approximately 20 Hz rhythmic activity that is transiently enhanced by median-nerve stimulation, a procedure discovered earlier [1,18].

Also in line with the association of M1 beta-band bursts and impaired movements, Gilbertson *et al*. [26] observed that movements are slowed down and the transcortical stretch reflexes are potentiated during increased 13–35 Hz activity in the M1 cortex. The authors interpreted these findings to imply preference for the existing motor and postural state, with new movements discouraged. The interpretation is in line with the original association of the rolandic mu rhythm to immobility [27]. Elegant further support for this *status quo* hypothesis was obtained from a repetitive TMS study where stimulation of the M1 cortex at 20 Hz, boosting 20 Hz oscillations in M1, slowed down movements [28]. A recent review on the functional role of beta-band oscillations also concluded that motor-cortex beta rhythms signal *status quo* [29].

After a median-nerve stimulus, which modulates rolandic rhythms, the levels of M1 beta-band oscillations and CMC (when the subject is keeping isometric contraction at least part of the time) covary closely [30]. Moreover, the M1 beta level and CMC are similarly related to the performance in a motor task, again implying a close connection [31].

#### (ii) Cortex–muscle coherence

In humans, CMC was first detected with MEG, occurring around 20 Hz during weak–intermediate isometric contraction [6,7] and around 40 Hz during strong isometric contraction [32,33]. Thereafter, the same phenomenon has been documented in a multitude of human MEG, scalp EEG and electrocorticographic studies, and it is well established in monkeys as well [34,35].

The early MEG recordings strongly implied the M1 cortex as the main cortical source of the coherence. For example, for foot versus hand muscle contractions, the source areas showed clear somatotopical order in the M1 cortex, with the maximum always in the contralateral hemisphere [7]. This finding of course does not rule out the CMC in other cortical regions, and in fact intracranial recordings have shown CMC, for example, in the pre-supplementary motor area (pre-SMA) and SMA proper [36,37]. However, we would like to emphasize that MEG–EMG coherence, as recorded in this study, is largely blind to SMA and predominantly reflects M1 activity.

Some of us [30,38] have previously advocated CMC as reflecting a mainly efferent flow of motor commands to the periphery. This view has been based on consistent findings about the cortical signals leading the muscular activity by about 20 ms to distal upper limb muscles and by about 40 ms to lower limb muscles. The time lag has been demonstrated by cross-correlograms between MEG and surface EMG signals, averaging of the MEG signals time-locked to EMG signals, and by phase spectra of the coherence in a wide frequency range covering the 20 Hz band (for reviews, see e.g. [30,38]).

However, the possible modulation of the CMC by peripheral input has been an issue in several recent studies and it could be hypothesized that the CMC modulation observed in this study during action observation might actually reflect somatosensory mirroring (for a review, see [39]), with the associated feedback from the body via somatosensory afferents. Below, we try to argue why this is not a viable explanation.

First, it is well established that the CMC—similar to the rolandic mu rhythm—disappears during changing force or finger displacement and then transiently rebounds after movement so that the rebound has been related to stabilization of the M1 cortex [8]. This interpretation is in line with a more recent hypothesis, already discussed above, that the corticospinal 13–35 Hz synchrony is related to a cortical state in which the postural set is reinforced and the speed of new movements impaired, thereby promoting the maintenance of the existing cortical state [26].

To further study the role of cutaneous afferents in CMC, Fisher *et al*. [40] applied local anaesthetic to produce digital-nerve block. Coherence between scalp EEG and surface EMG decreased during local anaesthesia and the finding was interpreted to support the role of peripheral (mainly tactile) afferents for the generation of the CMC. However, local anaesthesia made the task more difficult and the subjects' performance was deteriorated by increased jitter in the finger position. Thus, another interpretation for the findings is that the CMC was decreased because of the unintentional correction movements, as would be expected from the data of both Kilner *et al*. [8] and Gilbertson *et al*. [26]. Importantly, some subjects showed CMC after digital anaesthesia.

Pohja and Salenius [41] also addressed the role of peripheral afferents in the generation of CMC. They blocked peripheral sensory input with tourniquet ischaemia, assuming that the loss of sensory feedback should affect the CMC frequency. However, the CMC frequency did not change although the amplitude was reduced during ischaemia, suggesting that peripheral sensory feedback has a negligible role in the generation of CMC during stable isometric contraction.

We may thus conclude that any phasic change in the limb periphery modulates the CMC (easily abolishing it whenever the finger position or the force change) but during steady isometric contraction the MEG–muscle coherence can be considered a good index of the efferent population-level motor-command flow from the cortex to the spinal level.

#### (iii) M1 cortex and mirroring

Our current results on an increase in the CMC, with a peak about 500 ms after the onset of the observed phasic motor act and on a decrease in MEG power of ∼7 Hz and ∼15 Hz in an overlapping time window may seem contradictory given the previous considerations. Indeed, MEG power decrease would imply activation of the sensorimotor cortex [1], whereas increased CMC would indicate stabilization of the M1 cortex [8]. However, it is important to note that these two effects occurred at different frequency bands suggesting that different neuronal populations are involved in opposite functions at the same time. It is also evident from the source maps that MEG power was modulated statistically significantly in areas beyond the M1 cortex; the parieto-occipital suppressions likely reflected the visual effects of movement observation [14] and even somatosensory ‘mirroring’ [39,42,43]. As our current focus is in the motor-cortex output, we do not discuss these findings further.

M1 cortex is activated during observation of hand actions and even tool use [1,44]. However, it has been long under debate whether M1 is part of the mirror-neuron system as the initial monkey studies did not show (nor test) mirror-neuron activity in M1. One alternative interpretation thus was that as M1 is downstream from the inferior frontal gyrus (monkey F5 region), the core of the mirror-neuron system, it only passively reflects what has happened earlier in the processing chain.

Mirror neurons have now, however, been reported also in the M1 cortex of monkeys [45]. Roger Lemon's group first demonstrated that corticospinal-tract neurons originating from the monkey F5 area have mirror-neuron properties [46]. Some of these neurons showed suppressive activity during action observation despite increased firing during a monkey's own action; in other words, the concept of mirror neurons was now expanded from the previous definitions where mirror neurons were considered to react in a qualitatively similar manner during action observation and execution. The authors speculated that the suppressive action might be related to the inhibition of own imitative action during observation of movements.

More recently, the same group identified mirror neurons in the monkey M1 cortex [47]. Of all recorded M1 pyramidal-track neurons, half showed mirror-like activity. Most of these were increasing their firing during action observation (similarly as during action execution) but a large number of neurons also showed suppressed activity during action observation. The authors concluded that ‘M1 direct input to spinal circuitry is either reduced or abolished and may not be sufficient to produce overt muscle activity’.

Our current results, demonstrating a dual effect in the M1 cortex, can be interpreted within a similar framework as they imply that the human M1 cortex contains different neuronal populations, one of which (reflected in MEG power suppression) is automatically activated during observation of other persons' motor actions, and the other (reflected in CMC increase) is inhibited indicating stabilization of the M1 cortex; the latter effect could prevent the occurrence of inappropriate imitation or generation of any new movements associated with M1-cortex activation during action observation.

Anatomical and functional studies in monkey imply that cortico-motoneuronal connections—that comprise a part of all corticospinal connections, exist only in primates and are closely related to manual dexterity—predominantly emerge from the M1 cortex and not from the secondary motor cortices [48–50]. These M1-originated efferents could thus have a specific role in the inhibition of unwanted imitation during observation of other persons' actions despite the activation of the M1 cortex.
